# Medical Specialty Classification Based on Semiadversarial Data Augmentation

**DOI:** 10.1155/2023/4919371

**Published:** 2023-10-17

**Authors:** Huan Zhang, Dong Zhu, Hao Tan, Muhammad Shafiq, Zhaoquan Gu

**Affiliations:** ^1^Cyberspace Institute of Advanced Technology, Guangzhou University, Guangzhou, China; ^2^Department of New Networks, Peng Cheng Laboratory, Shenzhen, China; ^3^School of Computer Science and Technology, Harbin Institute of Technology (Shenzhen), Shenzhen, China

## Abstract

Rapidly increasing adoption of electronic health record (EHR) systems has caused automated medical specialty classification to become an important research field. Medical specialty classification not only improves EHR system retrieval efficiency and helps general practitioners identify urgent patient issues but also is useful in studying the practice and validity of clinical referral patterns. However, currently available medical note data are imbalanced and insufficient. In addition, medical specialty classification is a multicategory problem, and it is not easy to remove sensitive information from numerous medical notes and tag them. To solve those problems, we propose a data augmentation method based on adversarial attacks. The semiadversarial examples generated during the dynamic process of adversarial attacking are added to the training set as augmented examples, which can effectively expand the coverage of the training data on the decision space. Besides, as nouns in medical notes are critical information, we design a classification framework incorporating probabilistic information of nouns, with confidence recalculation after the softmax layer. We validate our proposed method on an 18-class dataset with extremely unbalanced data, and comparison experiments with four benchmarks show that our method improves accuracy and *F*1 score to the optimal level, by an average of 14.9%.

## 1. Introduction

Recently, deep neural networks (DNNs) have achieved remarkable success in classification tasks in various fields, such as computer vision [1], network anomalous behavior [2–4], and medical domain [5, 6]. The widespread use of electronic health record (EHR) systems has made the task of medical specialty classification become more important in modern healthcare. Classifying clinical notes into medical specialty fields improves the retrieval efficiency of the EHR system, which enables the doctor to quickly access the target information. In addition, automated medical specialty classification can be extended to other downstream applications, for example, assisting in medical knowledge extraction and supporting intelligent medical decision systems.

However, obtaining and labeling unstructured medical notes is not easy. Physician writing styles vary widely, as well as different probabilities of disease outbreaks in different medical subfields. These objective factors lead to existing datasets with significant deficiencies: insufficient data volume [6], nonopen access [5], and unbalanced categories [7]. Abundant medical specialty categories with little and unbalanced data are seriously impacting the performance of the classification model, which is the greatest challenge in the task of medical specialty classification.

As far as we know, the existing work focuses on how to design a more optimal model and tune the best parameters [6] to improve accuracy, such as comparing the effectiveness of different machine-learning models and deep-learning models, determining the best combination of models [7] or algorithms [5]. An approach of integrated data analysis was proposed in [5], where the researchers applied various techniques to extract features, including the unified medical language system and semantic network. However, the problems of insufficient and imbalanced data have been hardly considered in the existing work. In addition, the maximum number of categories considered in the available work is 9, less than the medical classification in medical specialty classification. A finer classification is more in line with the needs of realistic application scenarios, but it also implies a greater challenge.

Standing for the realistic scenario, we explore how to improve the performance of the classifier with the limited corpus. In this paper, instead of focusing on the model comparison and selection, we pay more attention to employing data augmentation technology which is an effective method to address the data imbalance problem. In the machine vision field, many outstanding augmentation techniques have been demonstrated to be effective in previous work [8]. However, for textual data, randomly modifying examples is ineffective due to the natural discrete nature of the text. In addition, data augmentation techniques applicable to different tasks vary widely, which leads to poor transferability.

To tackle these challenges, we developed a data augmentation method based on adversarial attacks. The adversarial attack aims to generate adversarial examples which are similar to the original examples but make model predictions wrong. From the geometric space perspective, the process of adversarial attacks is described as the process of clean examples approaching the decision boundary until it is completely crossed. Interestingly, the intermediate product of the attack process is identical to the definition of augmented data: data with a distribution close to that of the original data. Taking advantage of this property, we extend the training dataset using the intermediate examples generated in the attack process as augmented examples, which are called semiadversarial examples. Those examples better cover different regions of the decision space and improve both the generalization ability and robustness of the model. Furthermore, since nouns in medical notes play a key role in identifying the subfield to which the note belongs, we designed classifier architecture with confidence recalculation after the softmax layer by probabilistic information. This mechanism has advantages in supermultiple classification tasks, especially for categories with insufficient examples. Our contributions are summarized as follows.

1. We propose an adversarial-based data augmentation technique: SemiADA. It takes great consideration of the distribution of data in the decision space, which helps generate more comprehensive examples. Numerous experiments show that after employing the SemiADA method, the model accuracy and *F*1 score are improved by 15.1% and 14.7%, respectively.
2. We propose a weighted classifier with probabilistic information. Experimental results show that the proposed method proves to be excellent in medical classification tasks, especially in categories with insufficient examples.
3. We designed a medical specialty classifier based on a tough dataset situation. To the best of our knowledge, we cover the largest number of specialty categories. In addition, experimental results show that the classifier obtained by our method has stronger robustness.

## 2. Related Work

In this section, we explore existing related work in three areas: (1) classification tasks in the medical domain, (2) data enhancement methods, and (3) adversarial attacks as well as adversarial enhancement methods.

### 2.1. Medical Classification

Machine learning excels at classification tasks and plays an important role in smart healthcare. Image classification-related applications are particularly widespread. For example, in breast cancer detection, Fotin et al. [9] used AlexNet trained on a proprietary database to produce better performance than that achieved by years of engineering manual feature systems; in Alzheimer's detection, Lim and Schaar [10] utilized the flexibility and scalability of deep neural networks to enhance a joint longitudinal and temporal model of event data to predict the trajectory of Alzheimer's disease over time; in heart disease detection, Poudel et al. [11] introduced an RNN recursive connection in the U-net architecture to learn which information of the previous ventricle to remember when segmenting the next ventricle in a slice-by-slice segmentation of the left ventricle.

Compared to medical image classification, the application of machine learning to the medical classification of textual data has not been widely explored. For electronic health records, Weng et al. [5] constructed a machine learning-based natural language processing (NLP) pipeline and developed a medical subdomain classifier based on medical record content. Ahnaf et al. [6] used Bengali for training machine-learning and deep-learning models and used a bidirectional LSTM model to classify text-based records based on medical specialties. Cheng et al. [12] trained a CNN on a temporal matrix of medical codes for each patient to predict the onset of congestive heart failure (CHF) and chronic obstructive pulmonary disease (COPD).

### 2.2. Data Augmentation

Data augmentation techniques are proposed for solving insufficient data and poor data quality by constructing new examples to enrich the training data to improve the generalization ability of machine-learning models [13–15].

In terms of execution granularity, text data enhancement is classified into the character level, word level, phrase level, and document level. Character-level text data augmentation includes randomly changing a letter in a word [16], deleting or inserting characters [17], and modifying punctuation to induce weak text sounds [18]. Such methods have been shown to enable models to better handle noisy text. Phrase-level methods are based on structure [19] and interpolation [20]. This type of method is more restricted to specific languages and tasks. Common document-level methods include back translation [21] and generative methods [22].

The most widely promoted word-level approach is the text enhancement method based on synonym substitution [23, 24]. Embedding-based replacement aims at identifying more contextually appropriate words by using neural network embedding models and vector similarity calculation [25–27]. In contrast to plain synonym substitution, semantic and high-dimension-based methods take the context into account and have more comprehensive distributional assumptions. The BERT [28] model has been trained in a completion task with a large-scale corpus, making it capable of predicting [MASK] as a specific word. This feature of BERT is fully exploited in data augmentation techniques for word replacement, for example [18], proposed conditional BERT (c-BERT), which uses BERT contextual augmentation to generate augmented data.

Data augmentation techniques applied to the medical domain have focused on image enhancement. Janowczyk et al. [29] used SAEs to normalize H&E-stained histopathology images; Benou et al. [30] used CNNs to denoise DCE-MRI time series. Aydin et al. [31] combined images and text, using attention mechanisms and transfer-learning approaches to further improve medical data classification accuracy in small batches of data. In addition, methods based on GAN [32, 33] and reinforcement learning [34] are also used in image synthesis for the medical domain. Text-only data augmentation is difficult because label-preserving text transformations are hard to define [35, 36], and this disadvantage is accentuated in specific specialized fields, such as medicine.

### 2.3. Adversarial Attacks

Given a text *x*, the attacker adds imperceptible disturbance Δ*x* to *x* and aims to make the pretrained model *F* misclassify. Δ*x* operation includes adding, deleting, and replacing characters or words. In terms of textual form, there is some similarity between the adversarial and augmented examples in that they both generate similar copies of original examples by performing certain modification operations in the original example. In the natural language field, gradient-based adversarial training is effective in improving the accuracy and generalization of models [7, 21] but has weak gains in adversarial robustness. In addition, adversarial data augmentation [37, 38] and virtual adversarial data augmentation [21] also effectively improve the adversarial robustness of models, but such methods are prone to decrease model accuracy. Lee et al. [38] proposed a combination of friendly data augmentation and gradient-based adversarial training that can improve the adversarial robustness of models while maintaining their accuracy.

## 3. Methodology

### 3.1. Notions and Definitions

We denote *F* as the target model and *D*_orig_=(*x*_*i*_, *y*_*i*_)_*i*=1_^*n*^ as the original dataset. *x*_*i*_ is the text, denoted as the set of words *x*_*i*_={*w*_1_, *w*_2_, ..., *w*_*m*_}, and *m* is the number of words. *y*_*i*_ is the label of *x*_*i*_, and *y*_*i*_ ∈ *Y*, where *Y* is the set of all labels. *F*_*y*_(*x*) is the confidence (probabilistic score outputted by the softmax layer) of *F* predicting *x* as *y*. *F*(*x*) is the predicting label of *x*.

An adversarial example *x*_adv_ is generated by implementing imperceptible perturbations on *x* and indicated as *x*_adv_={*w*_1_′,  *w*_2_′, ..., *w*_*q*_′}. If attack methods are replacement-based, *m*=*q*.

The dataset after data augmentation is indicated as *D*_ada_. As for adversarial data augmentation, the steps are as follows: (1) train *F* on the original dataset *D*_orig_ to obtain a base model *F*_base_, (2) generate several semiadversarial examples {*x*_adv_′} for each text in *D*_orig_, (3) construct the adversarial dataset *D*_adv_={(*x*_adv_′, *y*_*i*_)}, and (4) train *F* on *D*_ada_=*D*_orig_ ⋃ *D*_adv_ to get the final model.

### 3.2. Semiadversarial Data Augmentation

Established data augmentation techniques fully consider how to enrich the training set by generating new data close to the original data but ignore the data distribution in the model decision space. The process of adversarial example generation well simulates the transformation of data location in the decision space. We presume that adversarial attacks can augment the dataset with a more comprehensive distribution. Although adversarial data augmentation has been shown to hurt model performance [39], perturbed examples that do not cross decision boundaries can overcome this drawback [40]. “Friendly adversarial examples” have been proposed and shown to improve the adversarial robustness of the model while maintaining accuracy [40]. Inspired by this, we propose semiadversarial data augmentation (SemiADA). Specifically, the multiple-step adversarial attack method (MSAA) generates semiadversarial examples for data augmentation. Semiadversarial examples are perturbed but do not successfully attack the target model. Multiple-step means we perturb several words for each attacking action.

**Figure 1:**
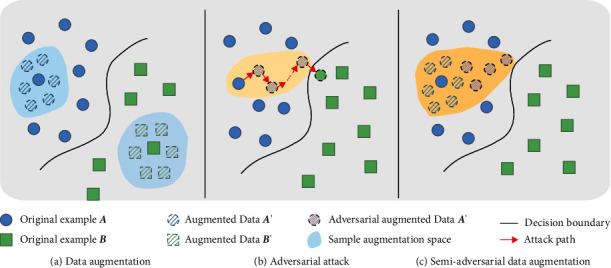
A visual illustrative example of (a) data augmentation and (b) adversarial attack. The circles and squares represent the different categories. The black curve represents the resultant decision boundary. As shown in the yellow-shaded part in (c), semiadversarial data augmentation covers a larger decision space.

A visual illustrative example is shown in Figure 1. Figure 1(a) describes the general data augmentation approach to generate semiexamples distributed around the original examples. The dynamic process of the adversarial attack is described in Figure 1(b). As shown in Figure 1(c), SemiADA can cover a larger area of the decision space. It is worth noting that there is a relatively large divide in the decision space between the perturbed and original samples as shown in Figure 1(c), but the texts are still highly similar to each other, which means perturbed examples reserve semantics.

In common attack algorithms, only one word or embedding vector is perturbed in each attack action, which is described as a single-step attack. Different from them, we propose a multiple-step adversarial attack method (MSAA), in which multiple words are selected as being perturbed in each attack action, and finally, a set of combined candidates are identified. During the MASS process, the semiadversarial examples generated in intermediate steps are retained as enhanced examples. Whole semiadversarial data augmentation is shown in Algorithm 1, which mainly consists of three steps as follows.

**Step 1 .**

*Wording Importance Ranking*. For any input *x*=[*w*_1_, *w*_2_, ..., *w*_*m*_], each word plays a different effect on the final prediction result. Therefore, we rank the importance of all words and perturb the important words in priority. Calculating the difference in confidence by deleting the word is a common way to compare words' importance. This type of method requires an access target model *m* times and is time consuming. To improve computational efficiency, we calculate the embedding vector difference of the replacement word as [MASK] and measure the importance of the word by the projection of the vector difference in the gradient direction. The importance of each word *w*_*i*_ in *x* is computed as *I*(*w*_*i*_, *x*):

(1) $$\begin{array}{c} I\left(w_{i},x\right)=\left(V_{\left[\text{MASK}\right]}-V_{w_{i}}\right)\nabla_{w_{i}}J\left(\theta ,x,f\left(x\right)\right), \end{array}$$

where the *V*_[MASK]_ is the embedding of [MASK], the *V*_*w*_*i*__ is the embedding of word *w*_*i*_, and *J* is the loss function of the model *F*. It only requires querying the model once to get the scores of all words, which greatly boosts efficiency.

We further filter out the stop words derived from NLTK (https://ww.nltk.org/) and Spacy (https://spcay.io/) libraries such as “the,” “then,” and “⋯.” Finally, we get the sorted and filtered set *W*.

**Step 2 .**

*Identify Candidate Word Combinations*. We construct a vocabulary dictionary by *D*_orig_, which contains 27816 words. We determine the synonym set Syn_*w*_*i*__ for each *w*_*i*_ in the dictionary, which is initiated with *k* closest words from the synonyms set of *w*_*i*_ by WordNet based on cosine similarity computation. WordNet [41] is a semantic-oriented English dictionary with 155,287 words and 117,659 synonyms. The word vectors used for similarity computation are from pretrained word embedding model Glove [42].

Human-written medical notes are not perfect and always contain some syntactic errors, so we do not need the generated augmented examples to be perfect. Unlike adversarial example generation, we aim to generate data that better meet the data augmentation conditions, so syntactic correctness checking is not strictly necessary.

In each attack action, we select the top *t* words from the sorted set *W* as the perturbed word set PerSet = *w*_*i*_, ..., *w*_*i*+*t*_ where *i* = *j∗t* and *j* is the *j*-th attack action. There are *k*^*t*^ kinds of all possible combinations, so it is extremely time consuming to try all replacements. To save overhead, we randomly example *r* = *k* × *t* times to reduce the number of combinations of exponential complexity by a constant value. The candidate substitution words are obtained as follows:

(2) $$\begin{array}{c} \text{CandiSet}={\left\{{\left\{R\left(w,\text{Sy}n_{w}\right),w\in \text{PerSet}\right\}}_{j}\right\}}_{j=1}^{r}, \end{array}$$

where *R*(*w*, *Syn*_*w*_) represents randomly selecting a word from Syn_*w*_.to replace *w*.

**Step 3 .**

*Construct Semiadversarial Examples*. We sequentially replace words in PerSet with the combination of candidate words in CandiSet to generate the perturbed examples *x*′. If the prediction probabilistic of *x*′ on the original label *y* is reduced, we add *x*′ to the final augmentation set. It is worth noting that we do not add the final adversarial examples to the augmented set because they mislead the decision boundaries of the model to deviate more from the true one. The idea that adversarial data augmentation leads to a decrease in model accuracy has also been experimentally verified in several works [39, 40].

#### 3.2.1. Complexity Analysis of Algorithm 1

According to the cyclical functions in the workflow, time complexity can be expressed as

(3) $$\begin{array}{c} T\left(n\right)=O\left(\frac{n}{t}\left(t+kt+kt\right)\right)=n+2kn=\left(2k+1\right)n. \end{array}$$

As *k* is a constant, the computation time is increasing as the input text size grows in a constant multiple. The time complexity of mainstream black-box adversarial attack methods tends to be above *O*(*n*^2^) [35, 43]. Benefiting from the idea of a multistep combinatorial attack in the attack (as shown in Step 1), our method is at least one rate lower than mainstream attack methods. We have confirmed it experimentally, as shown in Table 1.

### 3.3. Weighted Classification by Probabilistic Information

Data augmentation mechanisms considerably alleviate the problem of unbalanced and insufficient data, but the accuracy under supermultiple categories is still unsatisfactory. We focus attention on the task and the data itself to seek more solutions. In the medical field, nouns play an important role, and high-frequency words vary greatly across medical specialties. For example, the “stomach” often appears in the “gastroenterology” category but rarely in the “podiatry” category. We inferred that simple probabilistic statistical information is useful to express the differences between categories. Therefore, we considered incorporating probabilistic information (PI) for classification.

**Figure 2:**
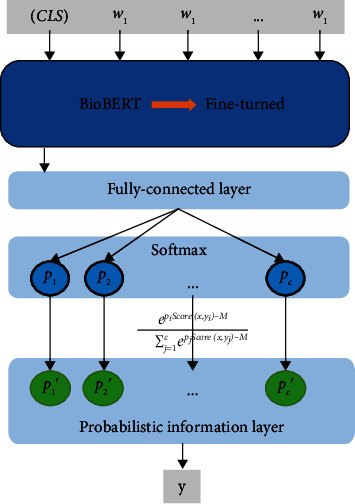
The classifier architectures of the proposed method. We add a probabilistic information layer to recalculate the probability distribution following the softmax layer.

We add the probabilistic information (PI) layer after the softmax layer (Figure 2). Its function is to recompute the probabilistic distribution and make the model prediction more accurate by incorporating the knowledge of probabilistic statistics. In the inference phase, for any input *x*, we perform the following steps.

#### 3.3.1. Calculating Word Category Importance

We propose the concept of word category importance (WCI) to indicate the relevance of different nouns to different medical specialties. Referring to the BM25 algorithm in information retrieval, we design the formula for WCI as

(4) $$\begin{array}{c} \text{WCI}\left(w_{i},y\right)={\text{IDF}}^{\prime }\times \frac{\left(k+1\right)\times {\text{TF}}^{\prime }}{k\left(1-b+b\left|D_{y}\right|/\text{avg}\left|D_{Y_{j}}\right|\right)+{\text{TF}}^{\prime }}, \end{array}$$

where |*D*_*y*_| denotes the total number of examples in the dataset whose labels are *y*, *D*_**Y**_*j*__ is the average data amount for all categories, and IDF′ is a variant of the inverse document frequency and expressed as

(5) $$\begin{array}{c} {\text{IDF}}^{\prime }\left(w_{i},y\right)=\log\frac{\left|D\right|}{\left|D_{w_{i}}\right|-\left|D_{y,w_{i}}\right|+1}+\left(1-a\right)\frac{\left|D_{y,w_{i}}\right|}{\left|D_{y}\right|}, \end{array}$$

where the damping factor *a* is a constant between 0 and 1 for restraining |*D*_*y*,*w*_*i*__|/|*D*_*y*_| contributions, |*D*|, |*Dw*_*i*_|, |*D*_*y*,*w*_*i*__|, and |*D*_*y*_| are the number of all texts in the dataset, the number of texts containing *w*_*i*_, the number of texts with the label *y* that contains *w*_*i*_, and the number of texts with the label *y*, respectively, and *TF*′ is the category frequency of a word, denoted as

(6) $$\begin{array}{c} {\text{TF}}^{\prime }=\frac{C_{y,w_{i}}}{C_{y}}, \end{array}$$

where *C*_*y*,*w*_*i*__ is the total times of the word *w*_*i*_ that appears in all examples with the label *y* and *C*_*y*_ is the total number of words in all examples with the label *y*.

#### 3.3.2. Estimating the Category Propensity of Input Examples

For any input *x*, we measure its propensity to belong to any category *y*_*i*_ based on the category importance of all words in *x*, denoted as

(7) $$\begin{array}{c} \text{Score}\left(x,y_{i}\right)=\sqrt{\frac{\sum_{w_{i}\in x}{\text{WCI}\left(w_{i},y_{i}\right)}^{2}}{n}}. \end{array}$$

#### 3.3.3. Probabilistic Distribution Recalculation

The softmax output of the target model is the normalized logit distribution, denoted as {*z*_*i*_|*i* ∈ *c*, ∑*z*_*i*_=1}, where *z*_*i*_ denotes the output of the *i*-th node and *c* denotes the number of categories. After the probabilistic information layer, the output of each node is

(8) $$\begin{array}{c} \text{Softmax}-\text{PI}\left(z_{i}\right)=\frac{e^{z_{i}\text{Score}\left(x,y_{i}\right)-M}}{\overset{c}{\underset{j=1}{\sum }}e^{z_{j}\text{Score}\left(x,y_{j}\right)-M}}, \end{array}$$

where *M*=max(*z*_*j*_Score(*x*, *y*_*j*_)_*j*=1_^*c*^) serves to prevent overflow of values.

## 4. Experiments

### 4.1. Experiment Setup

#### 4.1.1. Dataset

**Figure 3:**
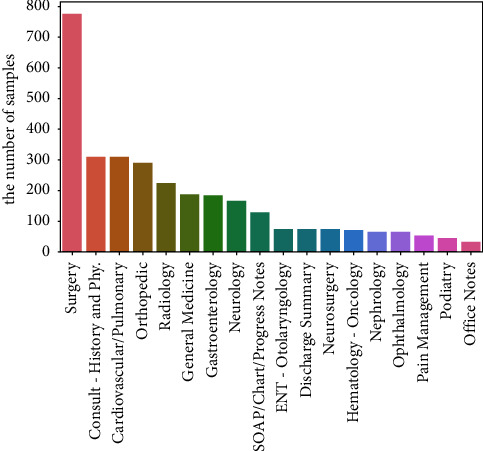
Data category distribution statistics. The horizontal coordinate indicates the categories, and the vertical coordinate indicates the total number of samples under that category. As we can see from the figure, data distribution is severely imbalanced.

**Figure 4:**
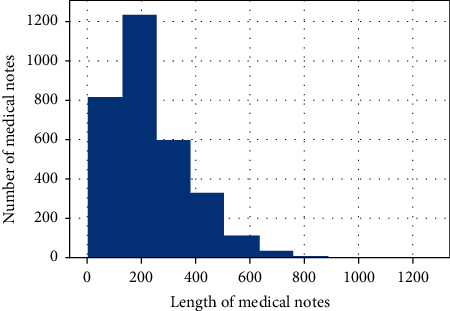
Text length statistics. The highest number of texts with lengths of 180–230 words. The horizontal coordinate indicates the total number of words in a note, and the vertical coordinate indicates the number of texts with a different total number.

We adopt the medical specialty classification dataset from Kaggle (https://www.kaggle.com/competitions/medical-specialty-classification/overview). The dataset of patient notes contains initial consultations, procedure visits, and so on. As some categories contain less than 30 items and are too difficult to train, we filter out the class where data numbers are less than 30. The filtered dataset includes 3,140 notes and 18 medical specialty categories. The distribution of the data is shown in Figure 3, and the distribution of text length after preprocessing is shown in Figure 4.

In the performance evaluation of different models trained in a plain way and the proposed method, we used stratified K-fold (*k* = 5). We divide the dataset into five folds and assign the training and test data in a 4 : 1 ratio. Data augmentation is processed for the training set only. Each metric score (Table 2) is derived from the average score of the test data of k-models. Considering the time consumption of data augmentation and retraining of a large model, in other experiments, we fix the test data and the training data, corresponding to the trained BioBERT model performs at the median in Table 2. In all training, the final training and validation sets are obtained by randomly dividing the training data in a 9 : 1 ratio in a stratified manner.

#### 4.1.2. Models

We adopt BioBERT as the classifier model. We utilize BioBERT with 12 transformer layers, 12 self-attention heads, and a hidden size of 768. We set dropout as 0.1, epochs as 10, max sequence length as 512, and batch size as 16. The learning rate of 1*e* − 5 is selected. In addition, we compare BioBERT with different models, including CNN, LSTM, and BERT. Specifically, the parameters of BERT are the same as those of BioBERT. The CNN model contains three convolutional layers with filter sizes of 3, 4, and 5. The LSTM model contains 2 bidirectional layers and 256 hidden units. We initialize them with 300-dimensional pretrained word embeddings Glove (https://github.com/stanfordnlp/GloVe) [42]. For both CNN and LSTM, the batch size is 64, the number of epochs is 16, and the dropout rate is 0.1.

#### 4.1.3. Evaluation Metrics

In this paper, we used accuracy, precision, recall, and *F*1 score to evaluate the performance of the model. Because the medical classification task is a multicategory problem, after the confusion matrix is formed by two categories, we average the confusion matrix to obtain the average of true positive (TP), false positive (FP), true negative (TN), and false negative (FN) as $\bar{\text{TP}}$, $\bar{\text{FP}}$, $\bar{\text{TN}}$, and $\bar{\text{FN}}$ and then calculate accuracy (Acc), microprecision (micro-*P*), microrecall (micro-*R*), and micro-*F*1 (micro-*F*1). The formulas expressions of all the used metrics are as follows:

(9) $$\begin{array}{c} \text{Acc}=\frac{\text{TP}+\text{TN}}{\text{TP}+\text{FP}+\text{TN}+\text{FN}},\\ \text{micro}-P=\frac{\bar{\text{TP}}}{\bar{\text{TP}}+\bar{\text{FP}}},\\ \text{micro}-R=\frac{\bar{\text{TP}}}{\bar{\text{TP}}+\bar{\text{FN}}},\\ \text{micro}-F1=\frac{2\times \text{micro}-P\times \text{micro}-R}{\text{micro}-P+\text{micro}-R}. \end{array}$$

#### 4.1.4. Experimental Environment

All models are trained in 4 GeForce RTX 2080 GPUs; the version number of the python environment used is 3.6.13; the model architecture used is the pytorch (https://pytorch.org/) library, and the version is 1.10.2.

### 4.2. Baselines

We utilize multiple data augmentation methods as comparison methods. The size of the augmented dataset is consistent. In addition to the examples in the augmented dataset, other training details are consistent.

#### 4.2.1. Plain Training

We use the dataset *D*_orig_ for plain training in four models without any extra optimization.

#### 4.2.2. Data Augmentation Based on Synonym Replacement (SRA)

All nouns, adjectives, verbs, and adverbs in text are replaced randomly with their synonyms based on WordNet [41].

#### 4.2.3. Data Augmentation Based on Embedding Replacement (ERA)

According to the replacement method described in [27], replacement is determined by two factors: whether the vector cosine similarity is less than the threshold and whether the lexical identity is consistent. Keeping the same experimental conditions as in the original paper, the threshold size is set to 0.7 in the experiments, and the NLTK library is used for lexical annotation.

#### 4.2.4. Data Augmentation Based on the Language Model (LMA)

We choose conditional BERT as the augmented language model [46], and the specific implementation follows the original algorithm scheme: randomly mask *k* words and then predict label-compatible words of the masked position and generate multiple new examples by replacing the predicted words. The value of *k* is 20% of the total number of words in the input examples.

#### 4.2.5. Adversarial Data Augmentation (ADA)

We use the adversarial examples generated by advanced adversarial example attack method Textfooler [43] as augmented examples based on TextAttack (https://github.com/QData/TextAttack).

### 4.3. Main Results

We investigated the effect of different models on the generalization ability of the models using the method proposed in this paper, and the results are shown in Table 2. We observe that the performance of BoiBERT and BERT models improves more than that of CNN and LSTM. Compared with the plain training of the four models, the BioBERT model pretrained with medical data has significantly better performance than other models.

As shown in Table 3, SemiADA + PI shows a significant improvement in performance in contrast to other augmentation techniques. It is worth noting that ADA leads to degradation in performance. The main reason for this phenomenon is that the augmented examples in ADA are adversarial examples that have led to changes in the labels and relatively large shifts in the decision boundaries of the model. In addition, SRA and ERA have comparable augmentation capabilities, and LMA performs better as it is based on the language model.

## 5. Further Discussion

### 5.1. Ablation Studies

We conduct ablation studies on the BioBERT model to clarify the impact of two parts of the proposed method. As shown in Table 4, SemiADA commendably improves the performance of the model in each metric, but precision is still higher than recall due to imbalance categories still existing. This issue can be well mitigated by the PI strategy. It is worth noting that although the PI strategy alone does not improve model performance significantly, it improves microrecall which means the classification accuracy of categories with small data size is improved.

### 5.2. Impact of Synonym Set Size

**Figure 5:**
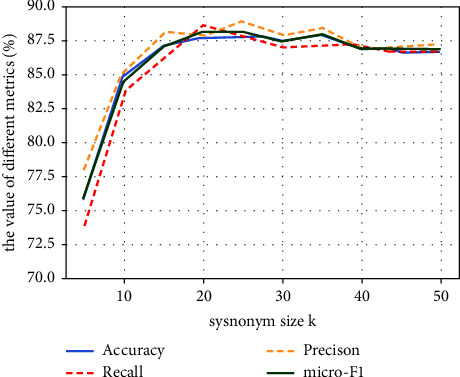
The performance of BioBERT with different synonym set sizes *k*. We select attacking step sizes of *t* = 3, 4, and 5 to conduct the repeated experiments, but only the results for *t*=3 are shown in the figure, as the performance shows the same trend in the three sets of experiments.

A larger synonym set size *k* means that there are more possibilities for word replacement and that more diverse augmented data can be generated. But does a larger *k* necessarily mean better performance? To further clarify the relationship between *k* and model performance, we slid *k* with a window size of 5 in the interval [5, 50] and observed the change in model classification performance. As shown in Figure 5, model performance does not significantly improve after *k* is greater than 20 and even has a slightly decreasing trend when *k* reaches 40.

### 5.3. Impact of Attack Step Size

**Figure 6:**
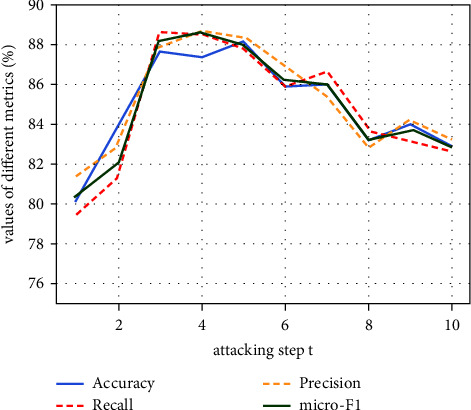
The effect of different *t* on the performance of the enhanced model.

We propose the MSAA method which perturbs *t* words in an attacking action for semiadversarial data augmentation. Larger *t* leads to a greater difference between the generated examples and the original examples, so there is less risk of the model falling into overfitting during the training phase. On the other hand, large *t* will make the semiadversarial examples to be very limited and insufficient to augment the dataset. We evaluated the effect of the generated augmented examples for different *t* ∈ [1,10], and the results are shown in Figure 6. We observe that the model works best for *t*=3. How to determine the value of *t* for different datasets in a more direct and automatic way needs to be further explored.

### 5.4. Impact of the Augmented Data Amount

**Figure 7:**
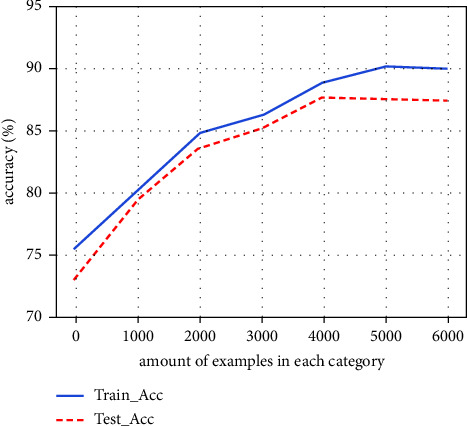
The accuracy of training and test phases with different amounts of augmentation data.

The appropriate number of augmented examples is important. An excessive number of augmented examples may lead the model into an overfitting dilemma. We compare the variation in training accuracy and testing accuracy of the models obtained by training different numbers of augmented texts. As shown in Figure 7, test accuracy no longer increases when the augmented data amount for each category reaches 5000.

### 5.5. Robustness Analysis

We evaluate the robustness of our method against four attack methods, which rely on the TextAttack library. Due to the inefficiency of the attacks for long text, we select 200 data for each experiment and repeat the experiment three times to take the average value. The maximum perturbation rate is set to 0.1, and the minimum text similarity threshold is set to 0.84. We summarize the robustness results of the plain training mode and our proposed method as shown in Table 5.

### 5.6. Visualization Analysis

To further verify our interpretation given in Figure 1, we compare the difference between SRA and SemiADA in the two types of vector representations: the difference in the embedding distribution on the hidden layer output of [CLS] position and the output of the softmax layer. The output embedding of [CLS] can be viewed as a sentence vector. The output embedding of the softmax layer is the most direct-viewing response to the distribution of examples in the decision space. Since the candidate words for both methods are derived from WordNet, the word vector distribution is the same, so we do not visualize and compare the word embeddings.

**Figure 8:**
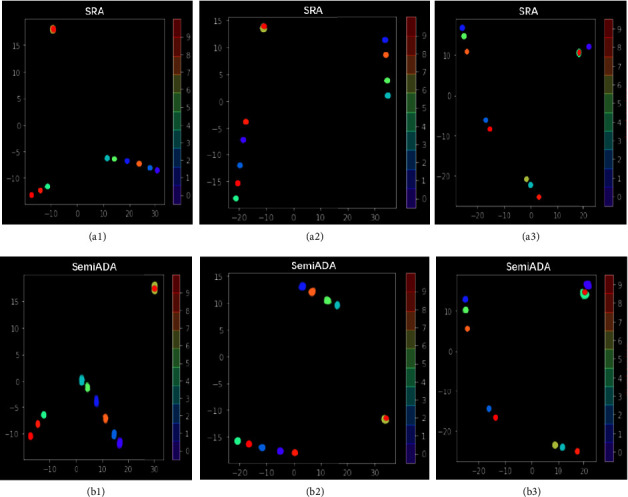
Comparison of sentence embedding distribution generated by SRA and SemiADA. We randomly select 10 original samples, which are sampled from different categories in the dataset. Then, we generate 20 new samples for each original sample by SRA and SemiADA. In order to avoid the overlap of embeddings from different categories, we add bias terms of different sizes to embeddings from different categories in the visualization, so that the categories are far away from each other. We repeat the experiment three times to obtain three sets of plots (each column is a set of experimental results), where the visualization results under the SRA method are shown in (a1–a3) and the visualization results under SemiADA are shown in (b1–b3). We only need to observe the coverage of each category (the area covered by each color). The larger the coverage means examples cover a wider range in the decision space.

**Figure 9:**
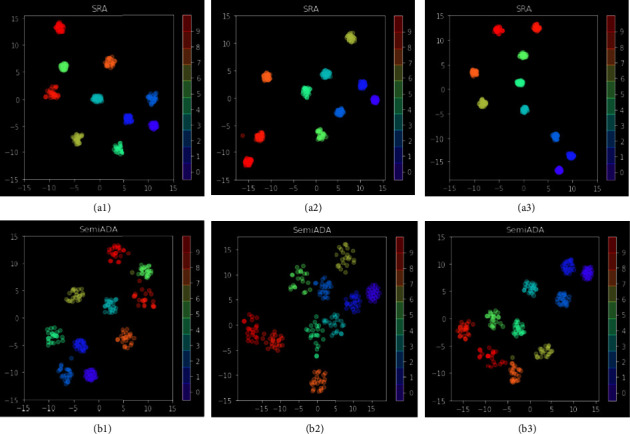
Visualization comparison for the output of the softmax layer. The samples used are exactly same as in Figure 8. (a1–a3) The visualization results under SRA and (b1–b3) the visualization results under SemiADA. It is easy to observe that the distribution of SemiADA is significantly wider (area covered by the same color).

Because both of those embeddings are high-dimensional vectors (768-dimensional, and 18-dimensional, respectively), we perform dimensionality reduction visualization by t-SNE. As we can see from Figure 8, although the candidate word distributions used by two methods are the same, sentence embeddings are markedly different from each other. The distribution of the new sentences generated by SRA is much closer to that of the original sentences (smaller area of the same color). As shown in Figure 9, in the decision space, the new samples generated by SemiADA are obviously distributed more scattered, while the samples generated by SRA are very close to each other. In other words, the new samples generated by SemiADA are richer and cover a wider area in the decision space. The candidate words have been strictly restricted to ensure that the data distribution of the new samples is unbiased. In this case, overly similar sentence distributions and extremely close inputs in the decision space can cause the model to fall into an overfitting state, which is the significant reason for the limited accuracy improvement.

### 5.7. Challenges and Limitations

Pretrained models are currently the most powerful tools for NLP as they significantly improve the accuracy of many NLP tasks and have strong generality. However, we also need to consider the resource consumption in model implementation, because the huge model architecture is not convenient for physical storage and application. We believe that lightweight models will be more popular in the medical industry, and this is the direction of our future research.

Healthcare is an important field regarding human life and development, with low fault tolerance for models and higher requirements for model interpretability. Dealing with vague and uncertain medical texts remains a challenging task. Literature studies [47, 48] give applications of fuzzy classifiers in key areas, which give us some insights. As fuzzy classifiers are transferable, we believe that the accuracy and stability of the models will be greatly improved by applying them to the healthcare domain.

Adversarial robustness aims to enhance security of the deep-learning model, and we have accomplished some throwaway work in this paper. We hope this will trigger more thoughts and exploration on the security and reliability of deep-learning model applications in the healthcare field.

## 6. Conclusions

In intelligent medical scenarios, training a high-quality model with nonideal data is an important task, which is the starting point of our work in this paper. We propose SemiADA, a data augmentation method based on semiadversarial attacks and probabilistic information, to address the problem of insufficient data amount and imbalanced data distribution in supermultiple classification tasks. Our approach significantly improves the performance of medical specialty classifiers in a cost-friendly manner. Experiments show that our proposed method performs significantly better than various data augmentation methods. In addition, the robustness of the model is evaluated under various attack methods. The results show our proposed method improves the adversarial robustness of the target model to a certain degree.

Our approach takes into consideration the idea of solving data problems in deep learning and the unique characteristics of data in the medical field, to complement each other and maximize performance gain. Such an idea is of great interest in cross-disciplines, such as the intersection of medicine and artificial intelligence, where this paper is positioned.

## Figures and Tables

**Algorithm 1:**
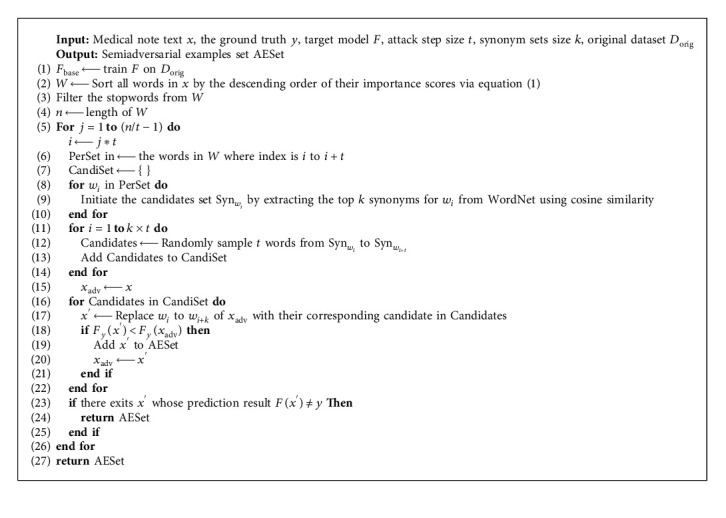
SemiADA.

**Table 1:** The performance of different attacks.

Attack	Accuracy	Time (minutes)	#Query
None	73.3	—	—
Textfooler [43]	20.1	47 mim	1207.12
Deepwordbug [44]	37.4	33 min	921.78
Textbugger [45]	40.6	40 min	947.50
BERT-attack [37]	17.2	67 min	1089.72
MSAA	40.5	12 min	240.91

The victim model is BioBERT trained on the medical specialty classification dataset. Time is execution times for each method that attacks BioBERT based on 1000 test examples by the plain way and the proposed method. #Query is the number of queries that methods require for the victim model. To make a fair comparison, MSAA is simplified by SemiADA that just generates adversarial examples but does not save the intermediate perturbed examples.

**Table 2:** The performance of different models trained in a plain way and the proposed method.

Models	Mode	Acc	Micro-*R*	Micro-*P*	Micro-*F*1
CNN	Plain	65.2	63.8	65.9	64.8
	SemiADA + PI	79.1	79.3	78.1	78.7

LSTM	Plain	70.5	70.6	71.9	71.2
	SemiADA + PI	81.6	84.1	81.2	82.6

BERT	Plain	69.4	71.5	69.6	70.5
	SemiADA + PI	83.9	83.9	83.0	83.4

BioBERT	Plain	73.0	71.5	74.7	73.1
	SemiADA + PI	**87.7**	**88.6**	**87.9**	**88.2**

SemiADA + PI is our proposed method, where SemiADA represents the data augmentation mechanism and PI represents the classification mechanism incorporating probabilistic information.

**Table 3:** The performance of different data augmentation modes on the BioBERT model.

Modes	Acc	Micro-*R*	Micro-*P*	Micro-*F*1
Plain	73.0	71.5	74.7	73.1
SAR	80.3	78.2	83.1	80.8
ERA	80.9	78.2	82.8	80.4
LMA	84.9	80.7	**88.1**	84.2
ADA	72.1	71.3	72.5	71.9
SemiADA + PI	**87.7**	**88.6**	87.9	**88.2**

Four metrics were used to compare the performance differences between the models: accuracy (acc), microprecision (micro-*P*), microrecall (micro-*R*), and micro-*F*1 (micro-*F*1).

**Table 4:** Ablation studies of our method on BioBERT.

Modes	Acc	Micro-*R*	Micro-*P*	Micro-*F*1
Plain	73.0	71.5	74.7	73.1
PI	73.4	78.1	77.3	77.7
SemiADA	85.6	83.2	86.9	86.9
SemiADA + PI	**87.7**	**88.6**	**87.9**	**88.2**

**Table 5:** The robustness experiment results of the plain training mode and SemiADA + PI training mode, including accuracy under attack (AUA %) and attack successful rate (ASR %).

Attacks	Plain		SemiADA + PI	
	AUA %	ASR%	AUA %	ASR%
None	73.0	—	87.7	—
Textfooler [43]	20.1	72.4	65.7	**26.2**
Deepwordbug [44]	37.4	48.6	59.6	**32.0**
Textbugger [45]	40.6	43.0	66.0	**24.9**
BERT-attack [37]	17.2	76.4	40.9	**53.6**

The black bold values denote the stronger robustness capability among two modes.

## Data Availability

The data we used can be found at https://www.kaggle.com/competitions/medical-specialty-classification.
